# Design and Investigation of PolyFermS *In Vitro* Continuous Fermentation Models Inoculated with Immobilized Fecal Microbiota Mimicking the Elderly Colon

**DOI:** 10.1371/journal.pone.0142793

**Published:** 2015-11-11

**Authors:** Sophie Fehlbaum, Christophe Chassard, Martina C. Haug, Candice Fourmestraux, Muriel Derrien, Christophe Lacroix

**Affiliations:** 1 Laboratory of Food Biotechnology, Institute of Food, Nutrition and Health, ETH Zurich, Zurich, Switzerland; 2 Danone Nutricia Research, Palaiseau, France; Colorado State University, UNITED STATES

## Abstract

*In vitro* gut modeling is a useful approach to investigate some factors and mechanisms of the gut microbiota independent of the effects of the host. This study tested the use of immobilized fecal microbiota to develop different designs of continuous colonic fermentation models mimicking elderly gut fermentation. Model 1 was a three-stage fermentation mimicking the proximal, transverse and distal colon. Models 2 and 3 were based on the new PolyFermS platform composed of an inoculum reactor seeded with immobilized fecal microbiota and used to continuously inoculate with the same microbiota different second-stage reactors mounted in parallel. The main gut bacterial groups, microbial diversity and metabolite production were monitored in effluents of all reactors using quantitative PCR, 16S rRNA gene 454-pyrosequencing, and HPLC, respectively. In all models, a diverse microbiota resembling the one tested in donor’s fecal sample was established. Metabolic stability in inoculum reactors seeded with immobilized fecal microbiota was shown for operation times of up to 80 days. A high microbial and metabolic reproducibility was demonstrated for downstream control and experimental reactors of a PolyFermS model. The PolyFermS models tested here are particularly suited to investigate the effects of environmental factors, such as diet and drugs, in a controlled setting with the same microbiota source.

## Introduction

The human colon harbors a large number of microbes forming a complex ecosystem responsible for various processes in the host. Under normal conditions, the gut microbiota acts as a barrier against enteropathogens, contributes to the development of the immune system and exerts important metabolic functions; which includes the production of short chain fatty acids (SCFA; such as acetate, propionate and butyrate) by breaking down complex carbohydrates that provide energy to epithelial cells and to the host [1, 2]. Each human harbors a unique gut microbiota composition consisting of bacteria belonging mainly to the phyla Firmicutes or Bacteroidetes and, to a lesser extent, to Actinobacteria, Proteobacteria and Verrucomicrobia [3, 4]. Colonization of the gut occurs first during birth, and throughout the first 2–3 years of life the microbial composition becomes established towards an adult-like microbiota. Recent studies indicate that the gut microbiota remains stable in adulthood, except for temporary alterations due to diet, disease and antibiotic treatment. However, an important shift in the microbial composition occurs during old age that is associated with a reduction in stability and often in biodiversity [3–7]. Additionally, a large inter-individual variability of the gut microbiota composition was reported for elderly Irish subjects of a large-scale *in vivo* study, with pyrosequencing reads assigned to the phyla *Bacteroidetes* and *Firmicutes* ranging from 3 to 94% [8]. To date, no common core microbiota for the elderly was defined, partly due to the various physiological factors, including lifestyle, diet and need for medications that change in old age. Thus, establishing the changes in the composition with ageing still require further investigations [4, 7].

Intestinal fermentation models allow the *in vitro* cultivation of gut microbiota to study their composition and function, uncoupled from the host. As such, models provide greater control, easier manipulation, and no ethical restrictions relative to *in vivo* studies, and are very complementary to *in vivo* strategies for elucidating mechanisms of gut microbiota [9]. Intestinal models have developed from batch for short-term fermentation studies to single or multistage continuous models that allow long-term studies due to substrate replenishment and toxic product removal [10]. However, one of the main challenges of the continuous culture models is the reproduction of the biofilm-associated microbes of the gut that is important to prevent washout of the less competitive bacteria. Immobilization of gut microbiota in gellan-xanthan gel beads has shown to reproduce the free and biofilm associated states of bacterial populations and to maintain the bacterial diversity at high cell densities in continuous intestinal reactors over periods of up to 71 days [10–12]. Furthermore, reproducibility and biological replication of continuous intestinal models was recently improved by the introduction of the PolyFermS model that allows the parallel testing of treatments with the same gut microbiota, and which has been validated for the child and the swine proximal colon [13, 14].

*In vitro* intestinal fermentations models have been developed and validated [10] to investigate factors of microbiota composition and metabolism of infants to adults while the elderly gut microbiota was only scarcely analyzed. Several studies were performed in continuous three-stage models for investigating the effects of antibiotics on *Clostridium difficile* infection. For these studies *C*. *difficile* was inoculated with mixed fecal samples from multiple elder donors and the system was challenged with antibiotics to promote the germination and growth of the sporulated bacteria, while microbiota analysis was only done with cultivation [15–18]. In a recent study batch cultures and continuous three-stage models inoculated with microbiota from single fecal samples of elder donors were used to investigate probiotics, prebiotics and synbiotics. Fluorescent *in situ* hybridization methods were used to monitor gut microbiota composition [19]. To date, no study has reported a detailed analysis of gut microbiota establishment and diversity in *in vitro* fermentation models reproducing the gut of aged (over 65 years) people.

The aim of this study was to investigate the use of immobilized fecal microbiota to develop different designs of continuous colonic fermentation models mimicking elderly gut fermentation. Immobilization of fecal microbiota obtained from three different donors was performed independently. Fecal beads were used to inoculate an immobilized cell reactor (IR) operated with conditions selected to mimic the proximal colon section of an elder. Three model designs, all starting with an IR used to generate a constant gut microbiota composition in proximal colon conditions, were tested for different experimental questions. These models were set in sequential order with the aim to investigate colonization of *Clostridium difficile* (data not shown in this paper). Model 1 was based on the three-stage design, with immobilized microbiota inoculated in a first proximal colon reactor connected to a transverse and a distal colon reactor, previously validated for infant and child microbiota fermentation [12, 20, 21]. Models 2 and 3 were developed based on the PolyFermS platform, which recently has been validated with child [13] and swine [14] microbiota, with adjusted conditions for the elderly microbiota. In model 2 IR containing immobilized fecal microbiota was used to inoculate (10% v/v) two parallel sets of 2-stage reactors mimicking the proximal and distal colon. Because *C*. *difficile* growth was only detected in distal colon reactors, in model 3 IR was used to feed (100% v/v) five reactors mounted in parallel, and mimicking conditions of the distal colon. This design allowed to test in parallel four treatments compared to a control in distal colon reactors. The microbiota composition in reactor effluents was monitored and compared to that of the corresponding fecal donor, and temporal stability of the models and reproducibility of downstream reactors within a PolyFermS model were demonstrated. Microbiota composition, diversity (qPCR and pyrosequencing) and activity (HPLC) were monitored in reactor effluents over operation periods of up to 80 days.

## Materials and Methods

### Ethics Statement

The Ethics Committee of ETH Zurich exempted this study from review because sample collection was not in terms of intervention. An informed written consent was, however, obtained from the fecal donors.

### Fecal inoculum and immobilization

For each fermentation experiment a fresh fecal sample from a different donor was used for the immobilization procedure. Fecal samples were collected from three healthy women, aged 71 (fermentation 1), 72 (fermentation 2) and 78 years (fermentation 3), who did not receive antibiotic treatment for at least three months prior to sample collection, and who did not consume probiotics on a regular basis.

Immediately after defecating, the fecal sample was transferred to a tube containing 5 mL of sterile, pre-reduced peptone water (0.1%, pH 7), placed in an anaerobic jar (Anaerojar, Oxoid, Hampshire, England), and transported and processed within three hours. Handling and encapsulation of the fecal microbiota into 1–2 mm gel beads composed of gellan (2.5% w/v), xanthan (0.25% w/v), and sodium citrate (0.2% w/v, Sigma-Aldrich Chemie GmbH, Buchs, Switzerland) was performed in an anaerobic chamber as previously described [21].

### Fermentation medium

The fermentation medium was based on the composition described by MacFarlane *et al*. [22] for simulation of adult chyme entering the colon. It contained (g L^-1^ of distilled water): pectin from citrus (2), xylan from oat spelt (2), arabinogalactan from larch wood (2), guar gum (1), inulin (1), soluble potato starch (5), mucin (4), casein acid hydrolysate (3), peptone water (5), tryptone (5), yeast extract (4.5), cysteine (0.8), bile salts (0.4), KH_2_PO_4_ (0.5), NaHCO_3_ (1.5), NaCl (4.5), KCl (4.5), MgSO_4_ anhydrous (0.6), CaCl_2_ x 2H_2_O (0.1), MnCl_2_ x 4H_2_O (0.2), FeSO_4_ x 7H_2_O (0.005), hemin (0.05) and Tween 80 (1). One mL of a filter-sterilized (0.2 μm pore-size) vitamin solution [23] was added to 1 L of autoclaved (20 min, 120°C) and cooled medium. All components of the nutritive medium were purchased from Sigma-Aldrich Chemie, except for inulin (Orafti®, BENEO kindly provided by RPN Foodtechnology AG, Sursee, Switzerland), peptone water (Oxoid AG, Pratteln, Switzerland), bile salts (Oxoid AG), tryptone (Becton Dickinson AG, Allschwil, Switzerland) and KH_2_PO_4_ (VWR International AG).

### Experimental setup

**Fig 1:**
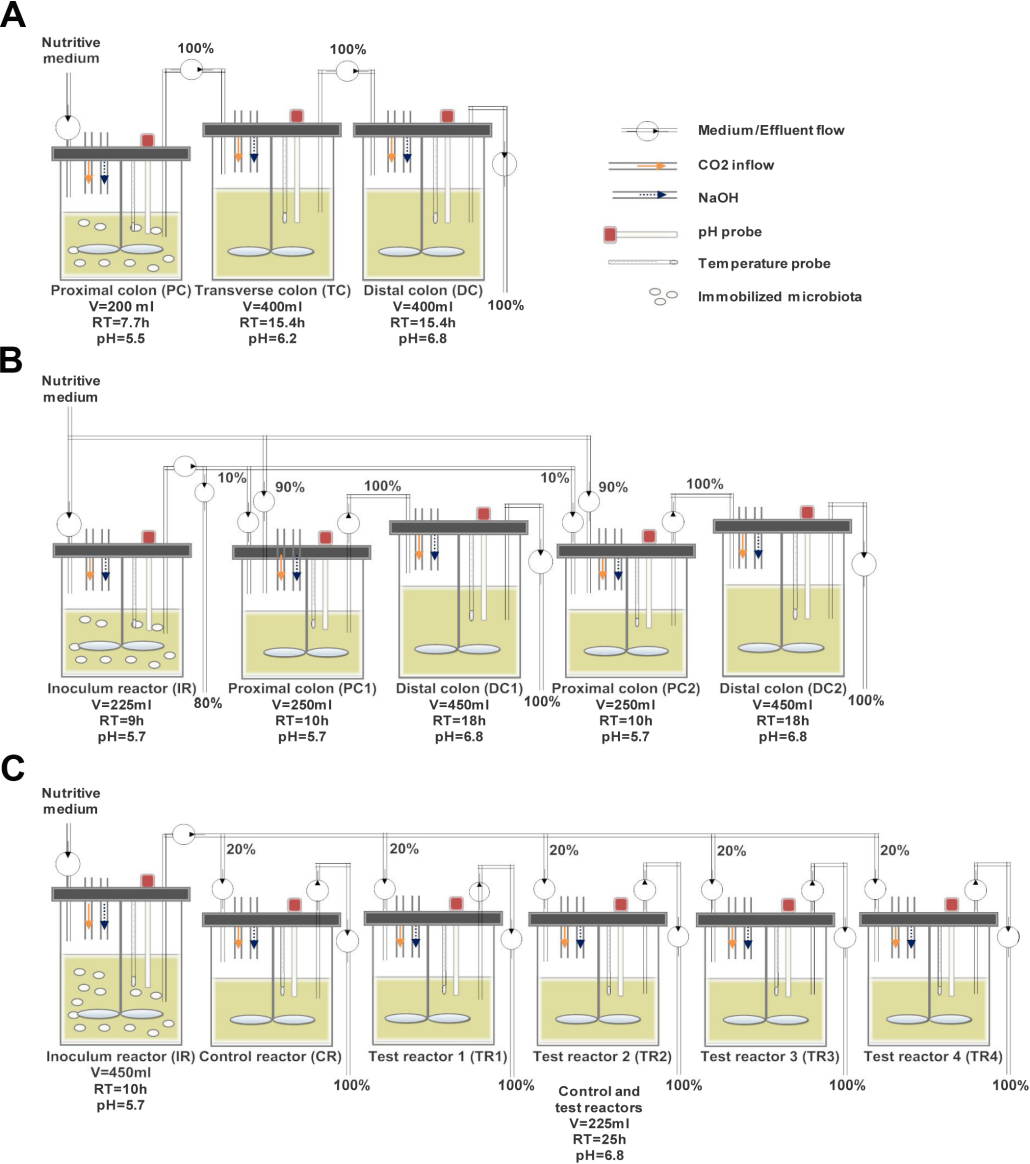
Set-up of the continuous fermentation models with immobilized gut microbiota. **(A) Model 1.** 3-stage model consisting of a proximal, transverse and distal colon reactor **(B) Model 2.** 2-stage model with an inoculum reactor connected to two parallel test systems consisting of a proximal and distal colon reactor **(C) Model 3.** 2-stage model with an inoculum reactor (proximal colon conditions) feeding 5 distal colon reactors connected in parallel; RT: Retention time, V: Volume.

Various reactor set-ups were applied for the three continuous intestinal fermentation experiments (Fig 1). The inoculum reactor (IR) seeded with donor’s microbiota immobilized in polysaccharide gel beads, and operated with proximal colon conditions, was common to all models. Model 1 was a classical three-stage system consisting of three reactors placed in series and operated under conditions of the proximal (PC corresponding to IR), transverse (TC) and distal colon (DC) [21]. Model 2 was based on the recently developed PolyFermS platform [13, 14] and consisted of an IR with immobilized fecal microbiota in proximal colon conditions used to continuously inoculate two parallel systems (10% v/v of the feed), each composed of a proximal (PC1 and PC2) and a transverse-distal reactor (DC1 and DC2). For model 3, the chyme medium fermented in IR with immobilized microbiota and operated with proximal colon conditions was used to continuously feed (100% v/v of the feed) five reactors mounted in parallel and mimicking conditions of a transverse-distal colon. In models 2 and 3, one system or reactor downstream to IR was used as control while the other system (PC2-DC2) or reactors (TR1-TR4) were used to comparatively test treatments, respectively.

### Fermentation procedures

The IR inoculated with 30% v/v gel beads was used to first colonize the beads with repeated-batch fermentations. The fresh medium was replaced every 12 h, for a total fermentation time of 60 to 72 h, depending on the model. Temperature was set at 37°C, stirring speed at 120 rpm and the pH was controlled at 5.5 or 5.7 by addition of 2.5 M NaOH. Sterile nutritive medium (4°C) was pumped continuously via a peristaltic pump (Reglo analog, Ismatec, Glattbrugg, Switzerland). Total mean retention times between 28 to 38.5 h were used in models 1–3, which is in the range of previously measured values for total colonic transit time in healthy elderly subjects that were between 25 and 66 h [24–28].

The reactors and nutritive media were continuously flushed with a low flow of CO_2_ to maintain anaerobic conditions during fermentation. The conditions of the models along with the model specific design and trials are briefly presented below and summarized in Fig 1.

#### Model 1

Six repeated-batch fermentations (72 h) were performed to colonize beads in PC (fermentation volume of 200 mL, pH of 5.5). Then two reactors mimicking conditions of transverse (TC) and distal colon (DC) (400 mL, pH 6.2 and 6.8, respectively) were connected in series to PC. The feed flow rate of the nutritive medium was set at 26 mL h^-1^, giving mean retention times of 7.7 h for IR and 15.4 h for TC and DC, for a total system retention time of 38.5 h. The model was stabilized for 14 days.

#### Model 2

Five repeated-batch fermentations (60 h) were performed to colonize beads in IR (225 mL and pH 5.7). The model was then switched to continuous mode with a medium flow rate of 25 mL h^-1^ for an additional five days. Two proximal colon reactors (PC1 and PC2, 250 mL and pH 5.7) were attached to IR, and each PC was connected to distal colon reactors (DC1 and DC2, 450 mL and pH 6.8) used to mimic transverse and distal colon conditions. Compared with model 1, the pH control set-point was increased from 5.5 to 5.7. This modification was implemented in order to better match the pH of the proximal colon section *in vivo*, which has been reported to be in the range from 5.5 to 5.9 [29] and to enhance metabolic activity in IR and PC’s. PC1 and PC2 were continuously inoculated with 2.5 mL h^-1^ (10%) fermented medium from IR and 22.5 mL h^-1^ (90%) fresh nutritive medium, for total flow rate of 25 mL h^-1^. Mean retention times in PC and DC reactors were 10 and 18 h, respectively, for a total retention time of 28 h (PC1 and DC1; PC2 and DC2). The model was continuously operated for an additional 18 days to reach stability and used for testing, for a total fermentation time of 55 days.

#### Model 3

Beads colonization was carried out in five repeated-batch cultures (60 h) in IR (450 mL and pH 5.7). Five DC reactors (225 mL and pH 6.8) mounted in parallel and mimicking conditions of transverse-distal colon were connected to IR. The fresh nutritive medium flow rate in IR was set at 45 mL h^-1^, while each DC reactors was fed with 9 mL h^-1^ medium fermented in IR. The mean retention times in IR and DC reactors of model 3 were 10 and 25 h, respectively, for a total retention time of 35 h. The model was stabilized for 14 days and used for testing, for a total fermentation of 80 days.

### Sampling and analysis

During continuous fermentations, effluent samples (10 mL) were collected daily from each reactor. Because the different models were also used for experimental trials with *Clostridium difficile* and antibiotic and probiotic treatments, only samples obtained during periods of control conditions are reported for model assessment. Analyses of microbial composition by quantitative polymerase chain reaction (qPCR) and pyrosequencing (model 2 and 3) were performed on samples from three days at the end of stabilization: days 9, 11 and 13 of model 1, days 16, 17 and 18 of model 2 and days 10, 11 and 12 of model 3. Metabolite concentrations in effluents of all reactors were tested daily during the entire fermentation by high-performance liquid chromatography (HPLC). Data corresponding to control condition periods (no treatment applied) are reported. Long term temporal stability was tested with IR data from models 2 and 3 since these reactors was not subjected to any manipulation over the entire culture period.

### DNA extraction

For qPCR and pyrosequencing analyses total microbial DNA of 200 mg feces and 2 mL effluent samples was extracted using the FastDNA® SPIN Kit for Soil (MP Biomedicals, Illkirch, France) and a final elution volume of 100 μL. DNA concentrations were determined using a Nanodrop® ND-1000 Spectrophotometer (Witec AG, Littau, Switzerland).

### qPCR analysis

Total bacteria and predominant bacterial groups were enumerated using specific primers (S1 Table). One μL of 10- or 100-fold diluted DNA was amplified in a total volume of 25 μL as described in [21], using 2 x SYBR Green PCR Master Mix (Applied Biosystems, Zug, Switzerland). Each reaction was run in duplicate on an ABI PRISM 7500-PCR sequence detection system (Applied Biosystems). For quantification, standard curves were produced by amplification of the DNA of the reference strain of the respective target group [30].

### HPLC analysis

SCFA (acetate, propionate, butyrate,valerate, isobutyrate and isovalerate) as well as formate and lactate concentrations) in fermentation effluent samples from all reactors were determined by HPLC analysis (Thermo Fisher Scientific Inc. Accela, Wohlen, Switzerland) in duplicate [31]. Effluent supernatants were 2-fold diluted with sterile ultra-pure water and filtered directly into vials through a 0.45 μm nylon HPLC filter (Infochroma AG, Zug, Switzerland). The analysis was run at a flow rate of 0.4 mL min^-1^ using an Aminex HPX-87H column (Bio-Rad Laboratories AG, Reinach, Switzerland) and 10 mM H_2_SO_4_ as eluent.

### Microbiota profiling by 454 pyrosequencing

454-pyrosequencing analysis of total genomic DNA of fecal and effluent samples was carried out at DNAVision (Gosselies, Belgium). The V5-V6 hypervariable 16S RNA region was amplified using specific primers 784F (5’- AGGATTAGATACCCTKGTA-3’) and 1061R (5’-CRRCACGAGCTGACGAC-3’) [32]. The forward primer contained the sequence of the Titanium A adaptor and a unique barcode sequence. Pyrosequencing was carried out using primer A on a 454 Life Sciences Genome Sequencer FLX instrument (Roche Applied Science, Vilvoorde, Belgium) following Titanium chemistry. The data obtained was analyzed using the open source software package Quantitative Insights Into Microbial Ecology (QIIME), v1.7 [33]. Raw sequencing reads were filtered based on selected quality criteria such as: (1) no mismatch with the primer sequences and barcode tags; (2) no ambiguous bases (Ns); (3) read-lengths not shorter than 200 base pairs (bp) or longer than 1000 bp; (4) the average quality score in a sliding window of 50 bp not to fall below 25; (5) excluding homopolymer runs higher than 6 nt. Sequences that passed quality filtering were clustered into OTUs at 97% identity level using cd-hit [34]. Representative sequences (the most abundant) for each OTU were aligned using PyNAST and taxonomically assigned using Greengenes v_13_08 database. ChimeraSlayer was used to discard chimeric sequences, based on a reference data set of sequences [35]. This led to 8245 +/- 1924 (mean +/- SD) reads per sample. These phylogenies were combined with absence/presence or abundance information for each OTU to calculate unweighted or weighted UniFrac distances, respectively, using rarefaction of 7000 sequences per samples. Unifrac measures the phylogenetic distance between sets of taxa in a phylogenetic tree as the fraction of the branch length of the tree that leads to descendants from either one environment or the other, but not both [36, 37]. Weighted and unweighted Unifrac metrics were used to build phylogenetic distance matrices. Principal coordinates analysis (PCoA) was applied to the distance matrices for visualization. Alpha diversity (diversity within sample) was calculated using Shannon (evenness) indexes. All 454-pyrosequencing files have been deposited to the National Center for Biotechnology Information (NCBI) Sequence Read Archive (SRA) under accession number SRP053000.

### Statistical analysis

Statistical analyses of HPLC and qPCR data (log10-transformed) were performed using JMP 8.0 (SAS Institute Inc., Cary, NC). Data are expressed as means ± SD of three days at the end of the stabilization period of each fermentation model. For every model the qPCR and HPLC data were compared between the reactors using the nonparametric Kruskal-Wallis test. *P* values < 0.05 were considered significant. Monte Carlo permutation procedure was used to determine difference between proximal and distal colon using 999 permutations. Correlation between genus-level phylotypes and metabolites (acetate, propionate, butyrate, isobutyrate, isovalerate and valerate) were done in fermentation models 2 and 3. Analysis was done using R package “Microbiome” [38] using Spearman correlation. P-values were corrected for multiple testing using Benjamini–Hochberg. Resulting q values < 0.05 were considered as significant.

## Results

### Microbial composition of fecal microbiota

The composition of dominant bacterial groups in fecal donor samples was assessed by analyzing the 16S rRNA gene copy numbers of total and selected bacterial groups using qPCR. All bacterial populations tested were detected in fecal samples, except for the *Roseburia* spp./*E*. *rectale* group and *Methanobacteriales* that were below the detection limit in the fecal inoculum of model 1 (Table 1). Predominant bacterial groups of all three fecal samples were *Bacteroides* spp. and *Clostridium* Cluster IV within which *Faecalibacterium prausnitzii* was dominant. *Enterobacteriaceae*, *Lactobacillus* spp. and *Bifidobacterium* spp. belonged to the subdominant populations in all three fecal inocula.

**Table 1:** qPCR enumeration of bacterial groups in fecal inocula and effluent samples of models’ reactors at the end of the stabilization period.

	Total 16S rRNA gene	*Bacteroides* spp.	*Entero-bacteriaceae*	*Lactobacillus* spp.	*Bifido-bacterium* spp.	*F*. *prausnitzii*	*Clostridium* Cluster IV	*Roseburia* spp./ *E*. *rectale*	*Methano-bacteriales*
**Model 1**									
Donor^1^	11.4	9.3	7.7	7.6	6.6	8.7	8.7	ND	ND
PC^2^	10.6 ± 0.02^A^	7.1 ± 0.1^A^	9.4 ± 0.1^A^	7.5 ± 0.2^A^	8.2 ± 0.1^A^	5.4 ± 0.2^A^	6.7 ± 0.4^A^	ND	ND
DC^2^	10.5 ± 0.1^A^	9.5 ± 0.01^B^	8.2 ± 0.1^B^	8.0 ± 0.2^B^	8.3 ± 0.1^A^	7.4 ± 0.1^B^	8.1 ± 0.1^B^	ND	ND
**Model 2**									
Donor^1^	11.1	9.6	7.2	8.3	8.5	10.1	10.3	9.4	8.6
IR^2^	10.2 ± 0.2^A^	10.0 ± 0.2^A^	8.9 ± 0.1^A^	6.1 ± 0.1^A^	6.7 ± 0.1^A^	9.1 ± 0.6^A^	10.1 ± 0.2^A^	8.6 ± 0.3^A^	8.8 ± 0.1^A^
PC1^2^	10.3 ± 0.05^A^	10.0 ± 0.2^A^	9.1 ± 0.3^A^	6.1 ± 0.02^A^	7.7 ± 0.1^B^	9.2 ± 0.4^A^	10.1 ± 0.1^A^	8.6 ± 0.3^A^	7.0 ± 0.4^B^
DC1^2^	10.3 ± 0.2^A^	9.9 ± 0.1^A^	8.9 ± 0.1^AB^	6.2 ± 0.1^AB^	7.6 ± 0.1^B^	9.2 ± 0.2^A^	9.9 ± 0.1^A^	8.6 ± 0.2^A^	8.9 ± 0.04^A^
PC2^2^	10.3 ± 0.1^A^	10.0 ± 0.1^A^	8.8 ± 0.3^AB^	6.2 ± 0.1^A^	6.6 ± 0.1^A^	9.0 ± 0.6^A^	10.0 ± 0.2^A^	8.4 ± 0.3^A^	7.3 ± 0.3^B^
DC2^2^	10.1 ± 0.2^A^	9.8 ± 0.2^A^	8.7 ± 0.1^B^	6.3 ± 0.001^B^	6.8 ± 0.2^A^	9.0 ± 0.2^A^	9.9 ± 0.1^A^	8.3 ± 0.2^A^	8.9 ± 0.1^A^
**Model 3**									
Donor^1^	11.5	10.6	6.3	7.5	6.2	10.6	10.3	9.7	7.7
IR^2^	11.2 ± 0.03^A^	9.9 ± 0.1^A^	9.6 ± 0.1^A^	6.7 ± 0.1^A^	7.8 ± 0.2^AB^	10.2 ± 0.1^A^	9.9 ± 0.1^A^	8.8 ± 0.1^A^	7.2 ± 1.0^A^
CR^2^	11.1 ± 0.1^A^	10.0 ± 0.1^AB^	9.5 ± 0.1^A^	6.9 ± 0.1^AB^	7.6 ± 0.1^AB^	10.1 ± 0.1^A^	9.8 ± 0.1^A^	8.5 ± 0.2^B^	8.3 ± 0.04^A^
TR1^2^	11.2 ± 0.2^A^	10.1 ± 0.03^B^	9.5 ± 0.1^A^	7.0 ± 0.01^B^	7.5 ± 0.1^A^	10.1 ± 0.04^A^	9.8 ± 0.1^A^	8.5 ± 0.2^AB^	8.3 ± 0.04^A^
TR2^2^	11.2 ± 0.05^A^	10.1 ± 0.03^B^	9.5 ± 0.1^A^	7.0 ± 0.2^AB^	7.6 ± 0.2^AB^	10.2 ± 0.02^A^	9.9 ± 0.02^A^	8.6 ± 0.1^AB^	8.3 ± 0.2^A^
TR3^2^	11.2 ± 0.1^A^	10 ± 0.2^AB^	9.5 ±0.1^A^	7.0 ± 0.2^AB^	7.5 ± 0.2^AB^	10.1 ± 0.1^A^	9.8 ± 0.1^A^	8.5 ± 0.2^B^	8.3 ± 0.2^A^
TR4^2^	11.2 ± 0.1^A^	10.0 ± 0.1^AB^	9.5 ± 0.1^A^	6.8 ± 0.4^AB^	7.7 ± 0.2^B^	10.2 ± 0.1^A^	9.9 ± 0.2^A^	8.6 ± 0.3^AB^	7.9 ± 1.0^A^

PC, proximal colon reactor; DC, distal colon reactor; IR, inoculum reactor; CR, control reactor; TR, test reactor; ND, not detected

^1^Data are mean log_10_ copies 16S rRNA gene g^-1^ feces; samples were analyzed in duplicate.

^2^Data are mean log_10_ copies 16S rRNA gene g^-1^ fermentation effluent ± SD of three last days at the end of the stabilization period; samples were analyzed in duplicate. Values with different letters are significantly different within one model (P < 0.05)

**Fig 2:**
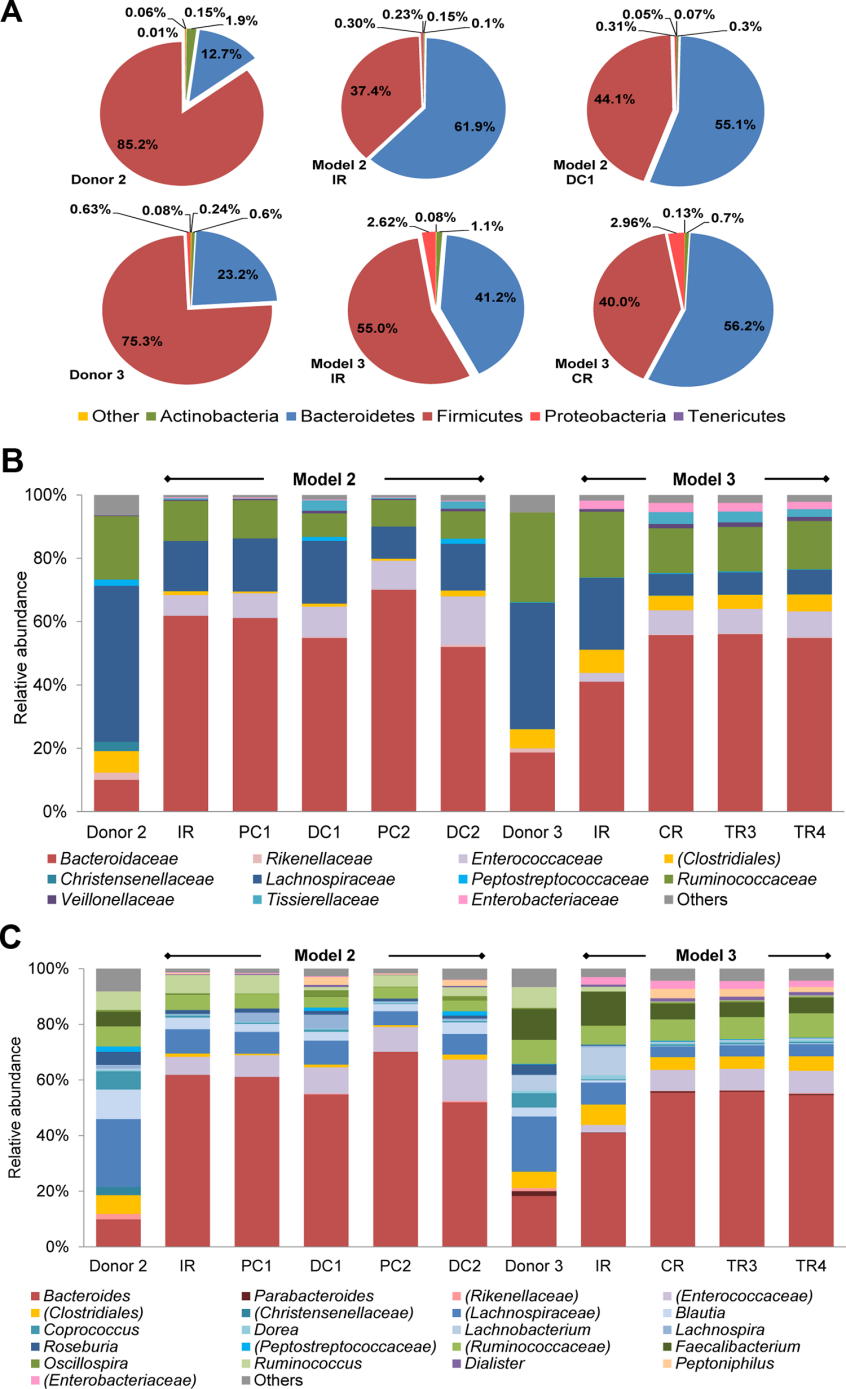
Microbial composition of fecal samples and reactors of PolyFermS models measured by 454 pyrosequencing. Relative abundance at **(A)** phylum level of fecal samples of donors 2 and 3, IR and DCI of model 2 and IR and CR of model 3 **(B)** family level and **(C)** genus level of fecal samples of donors 2 and 3, all reactors of model 2 and reactors IR, CR, TR3 and TR4 of model 3 identified by pyrosequencing of the V5-V6 hypervariable regions of the 16S rRNA gene. Effluent samples are average values of three last days at the end of the stabilization period. Parentheses indicate an unknown family belonging to an order or an unknown genus belonging to a family or order. Values < 1% are summarized in the group “others”.

The microbial profile of fecal inocula of model 2 and 3 was additionally analyzed by pyrosequencing (Fig 2). Phyla of both fecal samples were mainly assigned to Firmicutes and Bacteroidetes, with a Bacteroidetes:Firmicutes ratio of 0.15 and 0.30 for donor 2 and 3, respectively, and followed by Actinobacteria, Proteobacteria and Tenericutes (Fig 2A). The dominant families, *Lachnospiraceae*, *Bacteroidaceae* and *Ruminococcaceae* were similar for both microbiota (Fig 2B). At the genus level an unassigned genus belonging to the family of *Lachnospiraceae* was most abundant in fecal sample 2, followed by *Blautia* and *Bacteroides* (Fig 2C). In fecal sample 3 the same unassigned genus at similar abundance (∼ 20%) to fecal sample 2 was most abundant, closely followed by *Bacteroides*.

### Microbial composition of effluents determined by qPCR

Reactor effluents at the end of stabilization of all models were analyzed by qPCR to compare the microbial composition to the fecal donor and between reactors of a model (Table 1). Total bacterial numbers were, in general, high (> 10 log_10_ gene copies mL^-1^) but between 0.3 to 1.0 log_10_ lower compared to the corresponding donor’s fecal sample while no differences were observed between reactors within a model. In effluent samples of model 1, the *Roseburia* spp./*E*. *rectale* group and *Methanobacteriales* were not detected which was consistent with the lack of these groups in the corresponding fecal donor. *Bacteroides* spp. and *Enterobacteriaceae* were predominant in the PC and DC reactors of model 1, respectively. However copy numbers of specific population groups were significantly different between PC and DC effluents of model 1, with the exception of the total 16S rRNA gene and *Bifidobacterium* spp. The microbial composition of the DC reactor was more similar to the fecal donor than for the PC reactor, except for total bacteria, *Lactobacillus* spp. and *Bifidobacterium* spp.

Similar to the corresponding fecal samples, the predominant bacterial groups in effluents from model 2 and 3 comprised *Bacteroides* spp., *Clostridium* Cluster IV and *Faecalibacterium prausnitzii*. In model 2, no significant difference among all reactors was found for copy numbers of total 16S rRNA gene, *Bacteroides* spp., *Faecalibacterium prausnitzii*, *Clostridium* Cluster IV and *Roseburia* spp./*E*. *rectale* group. Only small (less than 0.4 log_10_) but significant differences were detected in DC2 for *Enterobacteriaceae* compared to IR and PC1, and for *Lactobacillus* spp. compared to IR, PC1 and PC2. *Bifidobacterium* spp. gene copy numbers were approx. 1 log higher in test system 1 compared to test system 2 and IR of model 2. *Methanobacteriales* numbers were significantly lower at PC conditions compared to IR and DC reactors.

In model 3, no significant difference was observed between reactors for most tested populations. Only small (≤ 0.3 log_10_ gene copies mL^-1^), but significant differences were measured for *Bifidobacterium* spp. numbers in TR4 compared to TR1, for *Bacteroides* and *Lactobacillus* spp. between IR and test reactors or CR, and for *Roseburia* spp. in CR and TR3 compared to IR.

### Microbiota profile and diversity in effluents determined by pyrosequencing

To assess the microbial diversity sequencing of the V5-V6 region of 16S rRNA gene was performed by 454 FLX pyrosequencing of all reactors effluent samples of model 2 and selected reactors (IR, CR, TR3 and TR4) of model 3 (Fig 2) at the end of stabilization phase and compared to diversity of corresponding feces. The main phyla in IR of model 2 and 3 were Firmicutes and Bacteroidetes followed by Actinobacteria, Proteobacteria and Tenericutes (Fig 2A). The ratio Bacteroidetes:Firmicutes was increased in IR and distal colon reactors of model 2 (ratios of 1.7 in IR and 1.2 in DC1) and model 3 (ratios of 0.8 in IR and 1.4 in CR) relative to the corresponding fecal inoculum (ratios 0.2 and 0.3, respectively). Bacteroidetes:Firmicutes ratios in DC2 of model 2 and TR3 and TR4 of model 3 were similar to DC1 and CR, respectively (data not shown). On the family and genus levels *Bacteroidaceae* and *Bacteroides* were dominant in all reactors of both models 2 and 3 (Fig 2B and 2C). *Bacteroidaceae* abundances increased from 10% to approx. 60% and from 36% to approx. 53% in the effluent samples of model 2 and 3, respectively, compared to the fecal donor samples. In general, very similar microbial patterns (family and genus level) were obtained for all reactors within a model. In both DC reactors of model 2 the abundance of *Bacteroidaceae* (54.8% for DC1 and 52.0% for DC2, Fig 2) decreased compared to PC reactors (61.2% for PC1 and 70.1% for PC2). Other small differences at family and genus levels between the PC and DC reactors of each test system were observed. In model 3, minor differences between composition in IR and DC reactors were observed while microbial patterns were highly comparable between the DC reactors.

**Fig 3:**
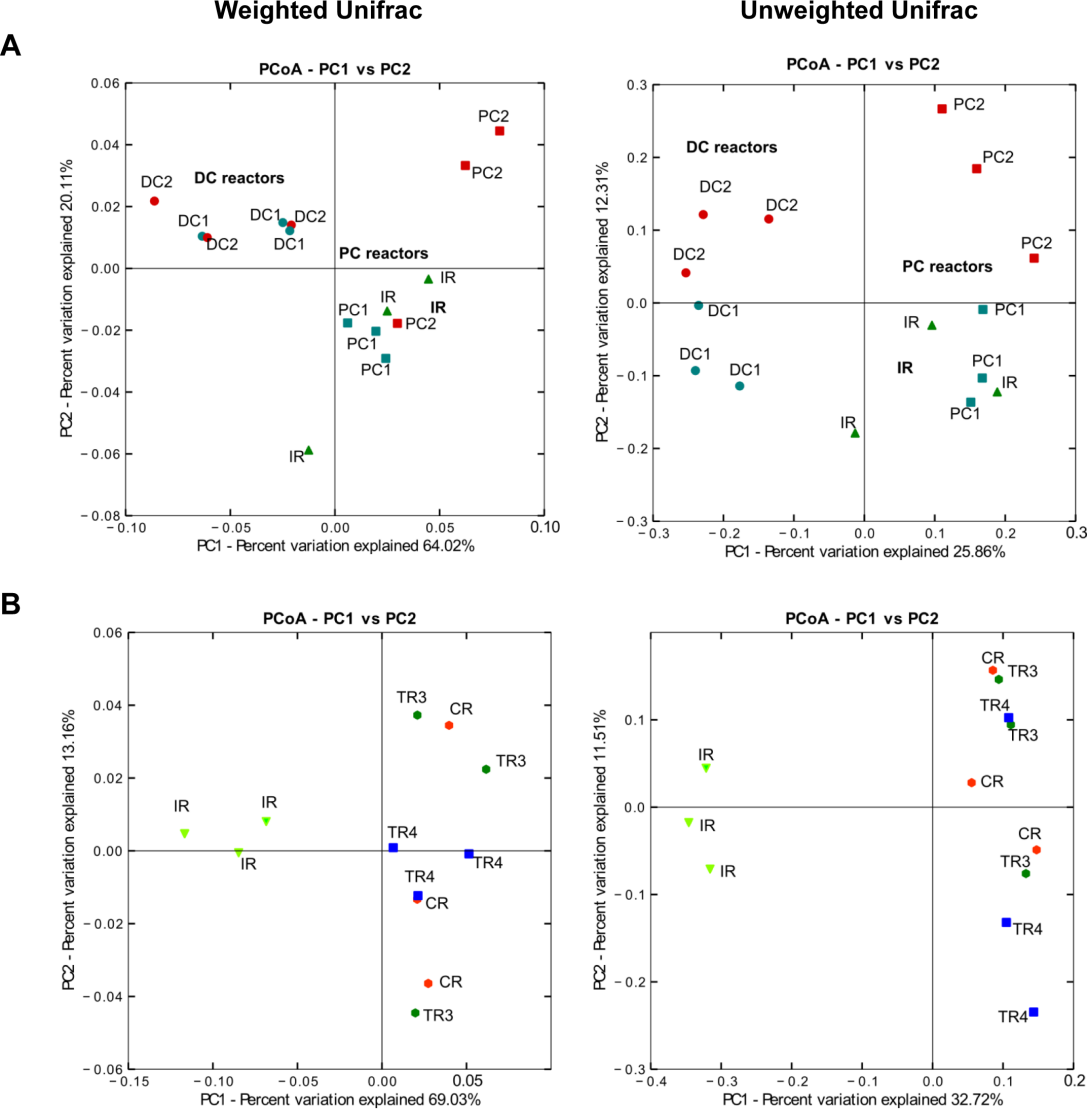
PCoA analysis of PolyFermS models based on weighted and unweighted UniFrac analysis. Each symbol is representing a different reactor. **(A)** Three last days at the end of the stabilization period of all reactors of model 2 (IR, PC1, DC1, PC2 and DC2) and **(B)** three last days at the end of the stabilization period of model 3 (IR, CR, TR3 and TR4).

Beta diversity (that measures the diversity between samples) of bacterial populations at the end of stabilization phase of model 2 and 3 was analyzed using Principal coordinate analysis (PCoA) (Fig 3). Significant differences between DC and PC were observed using both Unifrac distances (p<0.005). In models 2 and 3 a clear separation of reactors operated with proximal colon conditions (IRs, PC1 and PC2) and distal colon conditions (DC1- DC2, CR-TR3-TR4, respectively) was observed.

**Fig 4:**
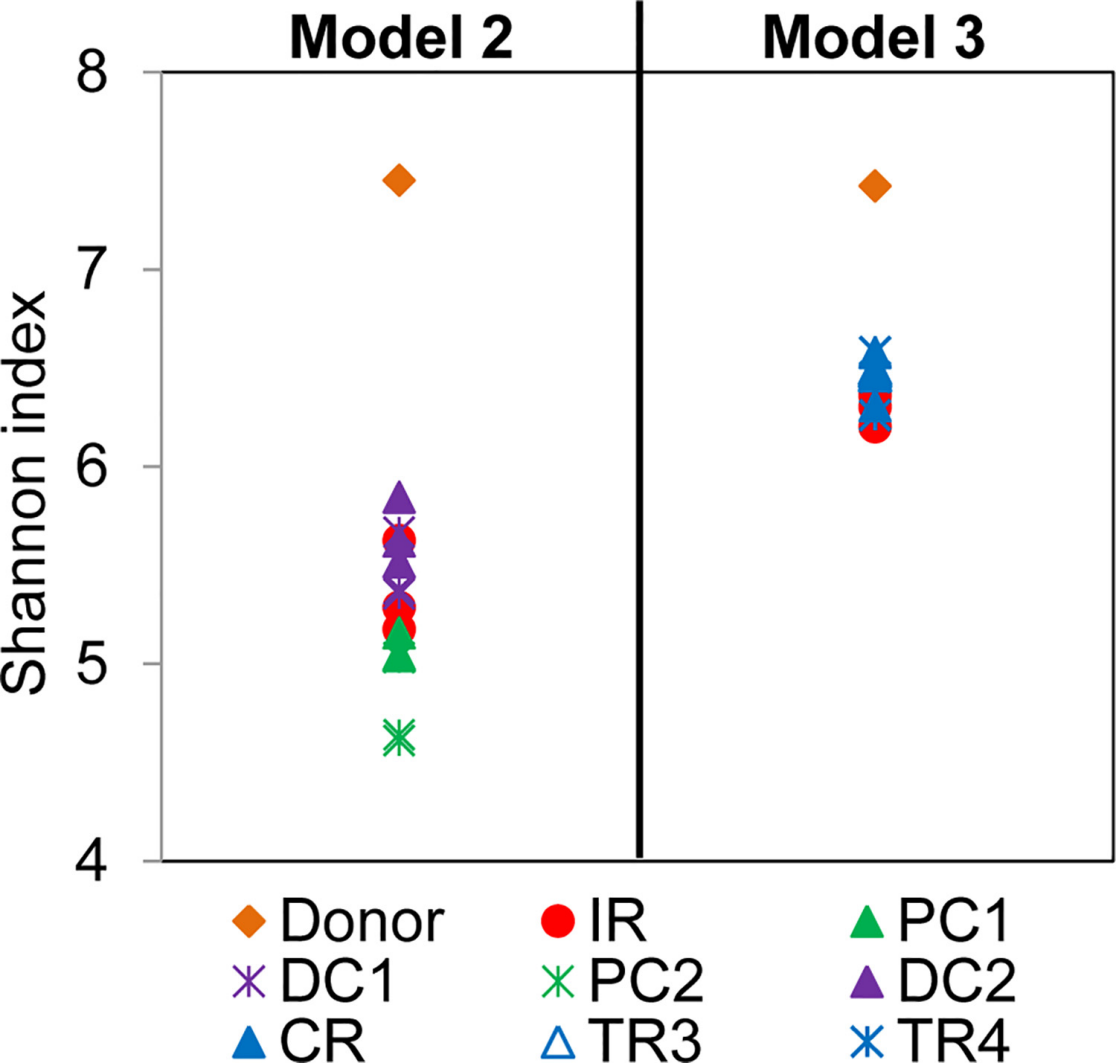
Shannon diversity index of fecal samples and reactors of PolyFermS models. The Shannon index was assessed in fecal donors 2 and 3, all reactors of model 2 and reactors IR, CR, TR3 and TR4 of model 3 of three last days at the end of stabilization phase. A higher Shannon index reflects a more diverse community (in abundance and evenness).

The Shannon diversity index was assessed for fecal donor samples and effluent samples of models 2 and 3 (Fig 4). A lower diversity was measured in model 2 (mean Shannon index of 5.3 ± 0.4 calculated for all reactors) and model 3 reactors (mean Shannon index of 6.4 ± 0.1 calculated for IR, CR, TR3 and TR4) compared to that of the corresponding fecal samples (Shannon index of 7.5 and 7.4 for model 2 and 3, respectively). In model 2, a higher diversity was obtained for DC (5.6 ± 0.2) compared to PC reactors (4.9 ± 0.2); while in model 3 the Shannon diversity was similar for all tested reactors with values between 6.2 and 6.6.

### Metabolic activity

**Fig 5:**
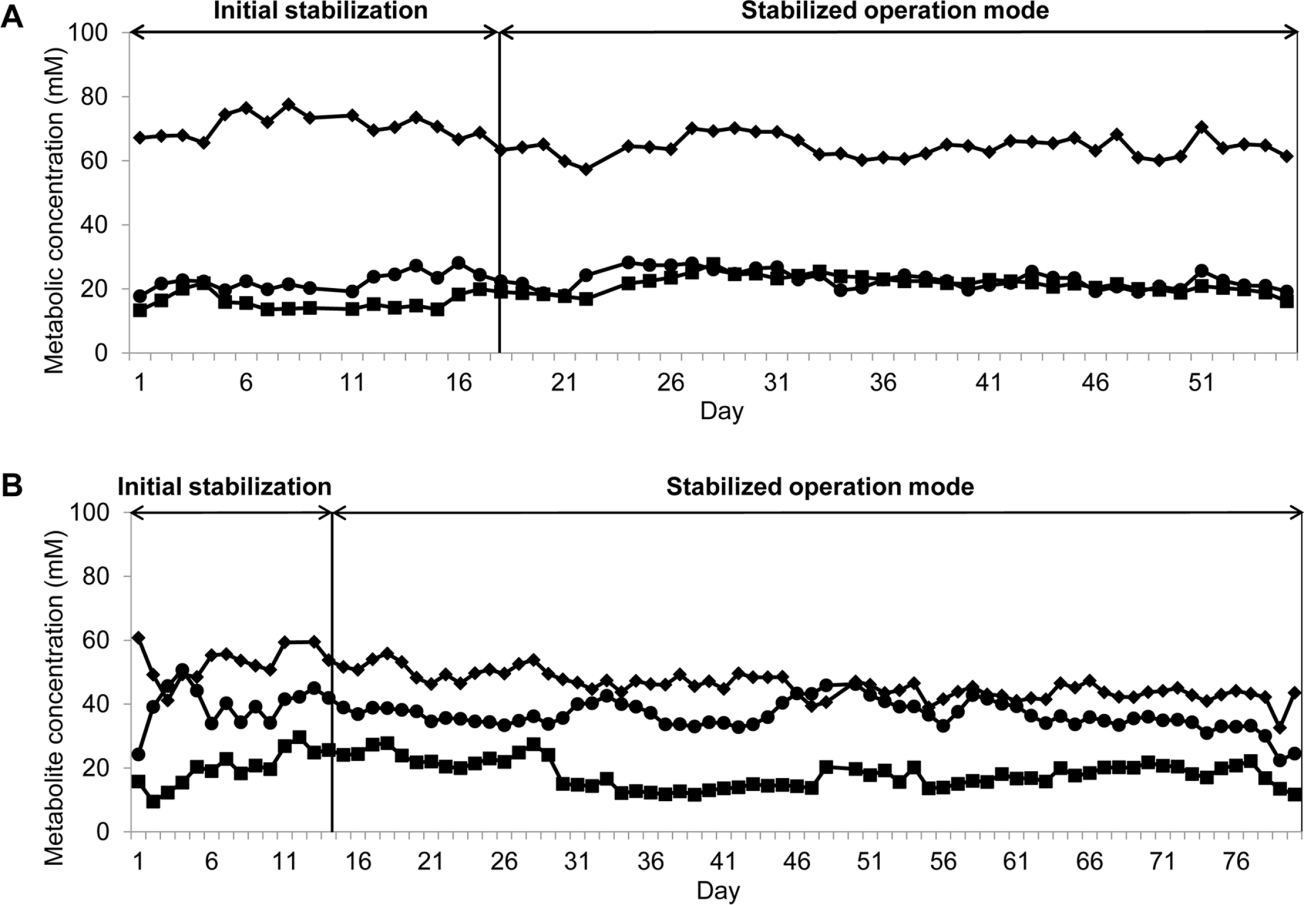
Daily mean SCFA concentrations in fermentation effluents of IR of PolyFermS models measured by HPLC. Initial stabilization: stabilization period in continuous mode to reach pseudo steady-state.; stabilized operation mode: continuous operation mode during pseudo steady-state conditions. **(A)** Model 2 and **(B)** model 3; (♦) acetate, (●) butyrate, and (■) propionate.

SCFA were measured by HPLC in fermentation effluents at the end of the stabilization period of all reactors of models 1, 2 and 3 to assess the metabolic activity and intra model stability (Table 2). After the initial stabilization periods, high and stable metabolic activities were measured over the entire fermentation in IR’s of models 2 and 3 which were operated under constant conditions and used to demonstrate temporal stability of the PolyFermS models (Fig 5).

**Table 2:** Metabolites concentration (mM) and ratios (%) measured by HPLC in effluent samples of models’ reactors at the end of the stabilization period.

Concentrations (mM)										Ratios (%)		
	Acetate	Butyrate	Propionate	Valerate	Isobutyrate	Isovalerate	Total SCFA	Acetate	Butyrate	Propionate	Valerate	BCFA
**Model 1**												
PC	74.1 ± 2.4^A^	5.4 ± 1.0^A^	5.4 ± 0.7^A^	ND	ND	ND	84.9 ± 2.7 ^A^	87.3	6.4	6.4		-
TC	108.6 ± 7.1^B^	15.4 ± 2.1^B^	15.3 ± 0.3^B^	ND	1.9 ± 2.1^A^	4.9 ± 1.2^A^	146.1 ± 7.4 ^B^	74.3	10.5	10.5		4.7
DC	110.7 ± 7.8^B^	15.4 ± 0.8^B^	15.3 ± 2.0^B^	ND	1.6 ± 0.3^A^	4.6 ± 1.5^A^	147.6 ± 8.1 ^B^	75.0	10.4	10.4		4.2
**Model 2**												
IR	66.3 ± 2.7^A^	25 ± 2.9^A^	19.1 ± 0.8^A^	ND	ND	ND	110.4 ± 4.0 ^A^	60.1	22.6	17.3		
PC1	69.1 ± 2.1^A^	20.8 ± 1.4^AB^	19.9 ± 0.6^A^	ND	ND	ND	109.8 ± 2.6 ^A^	62.9	18.9	18.1		
DC1	84.7 ± 2.5^B^	20.5 ± 1.3^B^	24.6 ± 0.2^B^	8.2 ± 0.2^A^	6.3 ± 0.2^A^	7.2 ± 0.1^A^	151.5 ± 2.8 ^B^	55.9	13.5	16.2	4.2	10.2
PC2	70.8 ± 2.9^A^	20.2 ± 3.3^AB^	26.5 ± 3.3^BC^	ND	ND	ND	117.5 ± 5.5 ^C^	60.3	17.2	22.6		
DC2	81.6 ± 1.1^B^	19.7 ± 2.6^B^	26.5 ± 1.8^C^	5.7 ± 0.2^B^	6.4 ± 0.2^A^	6.9 ± 0.4^B^	146.8 ± 3.3 ^D^	55.6	13.4	18.1	4.4	8.6
**Model 3**												
IR	56.5 ± 5.0^A^	40.2 ± 5.6^A^	23.7 ± 3.7^A^	ND	ND	ND	120.4 ± 8.4 ^A^	46.9	33.4	19.7		
CR	80.7 ± 4.5^B^	42.8 ± 1.8^A^	31.5 ± 2.4^BC^	ND	ND	0.8 ± 0.1^A^	155.8 ± 5.4 ^B^	51.8	27.5	20.2		0.5
TR1	76.8 ± 4.1^BC^	42.7 ± 5.0^A^	31.0 ± 1.7^BC^	ND	ND	ND	150.5 ± 6.7 ^BC^	51.0	28.4	20.6		
TR2	73.2 ± 1.9^C^	40.9 ± 1.3^A^	29.0 ± 0.9^B^	ND	ND	0.4 ± 0.1^A^	143.5 ± 2.5 ^C^	51.0	28.5	20.2		0.3
TR3	78.1 ± 1.1^B^	39.7 ± 1.4^A^	30.9 ± 0.9^C^	ND	ND	1.0 ± 0.8^A^	149.7 ± 2.0 ^B^	52.2	26.5	20.6		0.7
TR4	73.7 ± 0.7^C^	42.4 ± 1.9^A^	28.8 ± 1.1^B^	ND	ND	ND	144.9 ± 2.3 ^C^	50.9	29.3	19.9		

PC, proximal colon reactor; DC, distal colon reactor; IR, inoculum reactor; CR, control reactor; TR, test reactor

Data are means ± SD of three last days at the end of the stabilization period; samples were analyzed in duplicate. ND, not detected

Values with different letters are significantly different within one model (P < 0.05)

The concentrations of SCFA tested in reactors were model and reactor (-proximal and distal colon conditions) dependent, while intermediate products lactate and formate remained undetected in fermentation effluents. Acetate was the main metabolite in reactor effluents of all models, followed by butyrate and propionate which were generally produced at similar levels within a reactor. Butyrate concentrations were around 10 mM higher than propionate in CR and TR reactors of model 3. The molar ratios of acetate, butyrate and propionate in IR of the three models were different. In IR of model 1, operated at pH 5.5, a higher acetate fraction was produced (87/6/6) compared to IR of model 2 (60/23/17) and 3 (47/33/20) which were operated at a higher pH of 5.7. Higher concentrations of acetate and propionate were measured in TC and DC reactors of model 1, and DC reactors of models 2 and 3 compared to reactors IR and PCs of the same models, operated with proximal colon conditions. Butyrate concentrations increased along the reactors of the 3-stage model 1, but remained unchanged between PC and DC reactors of models 2 and 3. The mean concentrations of acetate, butyrate and propionate in the 5 distal reactors of model 3, were 76.5 ± 6.5, 41.7 ± 6.0 and 30.2 ± 3.4 mM, respectively, with small (less than 8 mM) but significant differences among reactors for acetate and propionate. Valerate was only detected in DC reactors from model 2.

Branched-chain fatty acids (BCFA) were not detected in any PC reactors. Isovalerate and isobutyrate were present in effluents samples from DC reactors of model 1 and model 2, at higher concentration in the latter. For model 3 isovalerate was only measured at low concentrations (≤ 1.0 mM) close to the detection limit in some DC reactors.

### Correlations between microbiota composition and metabolite production

**Fig 6:**
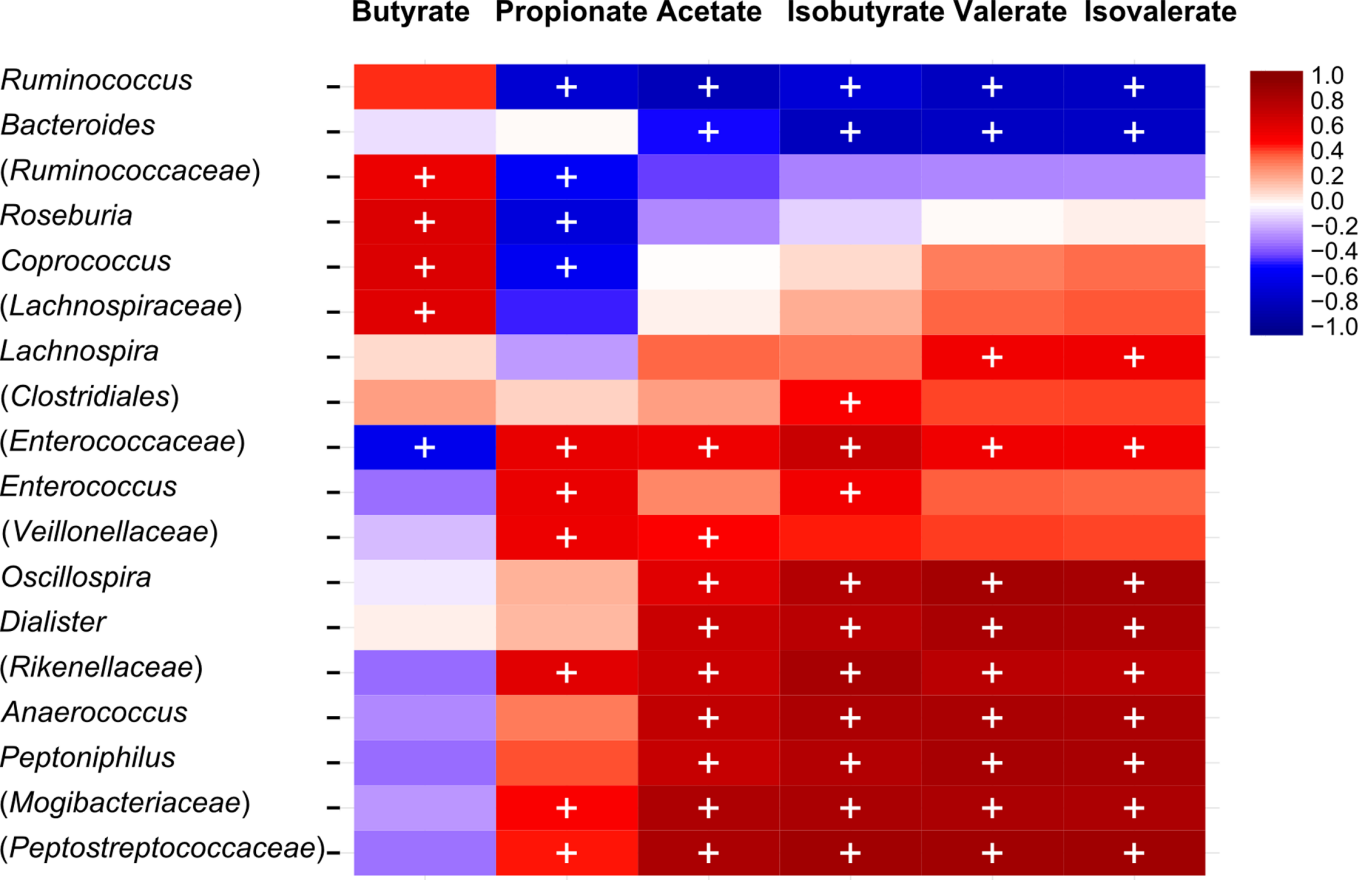
Correlations between genus-level phylogenetic groups and metabolites (SCFA, BCFA) of three last days at the end of stabilization period of model 2. The correlations, assessed by Spearman are indicated by either red (positive) and blue (negative), the significant correlations (q < 0.05) are indicated by ‘+’. Only genus related phylotypes > 0.1% and with at least one significant correlation with metabolites are depicted. Parentheses indicate an unknown genus belonging to a family or order.

Pyrosequencing data on the genus level and metabolite concentrations measured by HPLC were investigated to test significant correlations between phylogenetic groups and metabolic activity. For model 2, significant negative correlations were calculated between isobutyrate, isovalerate and valerate concentrations and the dominant genera *Ruminococcus* and *Bacteroides* (Fig 6). Butyrate was positively correlated with the dominant genus *Roseburia* and unclassified members of *Ruminococcaceae* and *Lachnospiraceae*. In contrast, a dominant unclassified member of *Enterococcaceae* was negatively correlated with butyrate and positively correlated with all other metabolites detected. Furthermore many genera detected at less than 1% (*Dialister*, *Anaerococcus* and unclassified genera of *Rikenellaceae* and *Mogibacteriaceae*) showed positive correlations with isobutyrate, isovalerate and valerate with the exception of *Oscillospira*, *Peptoniphilus* and an unclassified genus of *Peptostreptococcaceae* (with abundances above 1% but in distal colon reactors only). Correlations between metabolites (only acetate and propionate) and phylogenetic groups were also found for model 3 (S1 Fig).

## Discussion

Colonic fermentation models are useful tools to investigate factors that can influence the composition and metabolism of the gut microbiota, such as diet, antibiotic treatment, and bacterial infections *in vitro* and independent of the host [9, 10]. An important aspect for *in vitro* studies is the rational design of models and conditions, considering host target, model characteristics and limits, and the recognition that models are not perfect representation of reality. Therefore differences are often observed between fermentation samples and donor’s feces. A major discriminatory factor of *in vitro* models is the technique used for fecal inoculation. For most models, a fresh liquid fecal suspension is inoculated whereas this has been shown to lead to limited stability (washout of less competitive or slow-growing bacteria), cell density and difficulty to reproduce both the planktonic (lumen) and sessile (food particule and mucus associated) microbiota of the colon.

In the present study, we report the first-time investigation of continuous fermentation models with fecal microbiota obtained from different healthy volunteers aged between 71 and 78 years using in-depth characterization methods of the microbial diversity. We immobilized the fecal microbiota and inoculated the fecal biocatalysts in the inoculum reactor of the tested models with different designs. A dense and diverse microbiota could be established in PolyFermS models, with reproducible microbial composition and metabolic activity for downstream test and control reactors within a model.

During collection and immobilization of fecal microbiota from each elderly donor special attention was paid to keep anaerobic conditions from donor to reactor, in order to reproduce both the planktonic and sessile forms of bacteria in the colon, as previously suggested [10]. Gel beads can provide a protective microenvironment for the bacteria and allow the growth of complex and stable gut ecosystems at high cell densities of up to ca. log 11 cells per mL effluent as observed in the present study with elder gut microbiota, preventing the loss of slow growing bacteria. As expected from the lack of water reabsorption total bacteria numbers in reactor effluents of models were up to 1.0 log_10_ lower compared to the corresponding donor’s fecal sample. All bacterial groups tested in the fecal inoculum with qPCR were present in the corresponding models. The main differences in the bacterial composition and metabolic activity amongst models can be assigned to the different fecal inoculum used. In particular, *Roseburia* spp. was not detected in the feces and effluents of model 1 and this may explain the high acetate and low butyrate concentrations in this model (Table 2) since *Roseburia* is a main contributor for the conversion of acetate into butyrate [39, 40].

The pH of IR of model 1 was set to 5.5 in order to replicate the pH set in previous elder gut fermentation models [15, 17]. However, this pH is in the low range for the human proximal colon *in vivo*, [29, 41] and for models 2 and 3, the pH in IR and PCs was set to 5.7. This pH elevation induced an increase in total metabolites by approximately 20%, in agreement with previous observations of pH effect made in the PolyFermS model with child microbiota [13]. In contrast, butyrate concentrations did not increase in model 1 (pH 5.5) relative to model 2 and 3 (pH 5.7), as would have been expected from the stimulation of butyrate production at the lower pH, as observed in the previous study [13]. This is likely due to the lack of *Roseburia* spp. and the low *F*. *prausnitzii* numbers in model 1 which are the main butyrate producers in the human gut microbiota [42]. In models 2 and 3, the microbiota composition tested with qPCR was very similar between IR or PC reactors and distal reactors, while some limited changes were measured with pyrosequencing. In contrast, most targeted populations by qPCR significantly increased from PC (IR) to DC of model 1, suggesting that the low pH of 5.5 limited the growth of the targeted groups.

With qPCR we detected high *Enterobacteriaceae* copy numbers in reactor effluents compared to feces for all three models. This was observed in previous gut fermentation models [14, 21] and may be due to competitive advantage of these fast growing and robust bacteria that allows them to occupy niches during the immobilization process and the succeeding batch fermentation. The low levels of SCFA in the beginning of batch fermentation may further explain the increase in *Enterobacteriaceae* in reactors, as SCFA have inhibitory effects against *Enterobacteriaceae*, such as *Escherichia coli* [43].

The microbial composition of models 2 and 3 and corresponding fecal inocula may be considered more representative of the elderly population than the fecal inoculum of model 1, which did not harbor *Roseburia* spp., although the genus *Roseburia* was assigned at approximately 3% in fecal samples from elderly Irish subjects [8].

454 pyrosequencing was performed using the V5-V6 hypervariable region that was previously used to profile gut microbiota [44–46]. Sun *et al*. [47] recently reported that intragenomic heterogeneity for the V6 region may introduce overestimation of prokaryotes diversity. However in our study pyrosequencing data were used to compare of composition of donor and reactor samples within a model which should not be affected by this possible bias. In general, similar microbial profiles between effluent samples of model 2 and 3 and corresponding fecal donors were obtained. However, in both models the ratio of Firmicutes:Bacteroidetes was decreased when compared to the fecal sample, likely due to host-related factors including water and metabolite absorption and intestinal cells and host interaction, both of which are lacking in the fermentation models [13, 48]. Changes in microbiota composition and diversity may also reflect a possible loss of bacteria from donor to reactor, even though great care was taken to protect viability (strict anaerobiosis, mild conditions, short time), and the use of fecal microbiota to inoculate the inoculum reactor run in proximal colon conditions. Furthermore, the strictly controlled environmental factors, such as pH, transit time and medium composition in the *in vitro* models do no fully represent the specific donor conditions, thereby further contributing to *in vitro* and *in vivo* variations [10]. Despite the increase in Bacteroidetes in models 2 and 3, the Firmicutes:Bacteroidetes ratios of the fecal donor and the models were all in the range of previously reported data recorded during a large-scale *in vivo* study with elderly Irish people [8]. Indeed large inter-individual variations were observed in this study; however, the Bacteroidetes:Firmicutes ratio was shown to be higher in the elder, relative to the adult population. The majority of the reads was assigned to Firmicutes and Bacteroidetes while only low levels of Proteobacteria and Actinobacteria were detected in concordance with *in vivo* findings [8, 49]. Many of the predominant genera (abundance > 1%) including *Bacteroides*, *Faecalibacterium*, *Roseburia* and *Ruminococcus* were also found above 1% in elderly Irish subjects [8]. No in-depth characterization of the microbiota in donor and effluent samples was reported in previous investigations of fermentation models of the elderly microbiota, in which only traditional plating methods [15, 18] or FISH [19] were used.

SCFA are mainly produced from carbohydrate fermentation and to a lesser extent via degradation of proteins and amino acids; the effects of SCFA on the host are well documented [1]. It was previously found that the major SCFA found in stools of healthy volunteers between 14–74 years of age were: acetate, propionate and butyrate at an approx. ratio of 3:1:1 [50]. In our study, similar ratios were found in distal colon reactors of models 2 (4:1.5:1) and 3 (3:1.5:2), whereas in model 1 the acetate fraction was considerably higher (7:1:1), likely due to the low pH of 5.5 in IR and the fecal microbiota used in this model as discussed above. *In vivo* investigations are, however, hampered by the continuous absorption of metabolic products, which results in less than 5% of total production excreted in the feces [51] along with the difficulty associated with obtaining samples from different regions of the colon. Therefore, metabolite concentrations and ratios in feces are not indicative for the colonic microbiota activity. In contrast *in vitro* modeling allows accurate measuring of the metabolic activity of the gut microbiota for the tested model conditions. Stable SCFA concentrations were obtained throughout the fermentation in the untreated IR’s of the PolyFermS models demonstrating maintenance of gut microbial activity over the entire fermentation of 55 and 80 days for model 2 and 3, respectively (Fig 5).

BCFA are products of protein and amino acid fermentation but the formation of BCFA and associated species is not well studied [52]. In the colon of elders, an increase in proteolytic activity and a decrease in concentrations of SCFA were reported [53, 54]. Metabolites of amino acid fermentation can have toxic effects on the colonic lumen and were associated with several gut disorders [52]. In the tested models, BCFA were solely detected in significant levels within the distal reactors of model 1 and 2. This observation is consistent with the understanding that the distal colon is the major site for proteolysis whereas carbohydrate fermentation is the main energy yielding process in the proximal colon, resulting in a lower pH in this section [43, 55]. In model 2, genera with abundances of less than 1% were positively correlated with isobutyrate, valerate and isovalerate, suggesting that the dominant bacteria were mainly responsible for saccharolytic fermentation while proteins were degraded by the subdominant populations. Very low or no BCFA were detected in CR and TR reactors of model 3 which was set to mimic fermentation of transverse-distal colon sections within one reactor. This may be explained by the microbiota composition of model 3 that was different from model 1 and 2. The microbiota-dependent production of BCFA suggests the importance of using individual microbiota for inoculating intestinal fermentation models instead of pooling microbiota from different donors, as done in many studies for inoculation of gut fermentation models.

A major feature of the PolyFermS models over the three-stage model (model 1) is that several treatments can be investigated simultaneously and compared to a control inoculated with the same microbiota, thus generating reproducible and accurate data rather than when treatments are applied during consecutive periods. In both PolyFermS models microbial diversity and metabolic activity was very similar between control and test reactors. Model 2 built with two sets of proximal and distal colon reactors can be used for a broad range of studies, in proximal and distal colon conditions, such as the effect of an altered diet and administration of antibiotics on the gut microbiota in old age. Furthermore, the model is applicable for the *in vitro* investigation of the elderly microbiota in combination with health-related questions such as the manipulation of the gut microbiota using pro- and prebiotics [56]. PolyFermS model 3 built with multiple parallel distal colon reactors can be especially useful to study the effect of factors related to age on microbial metabolism in the lower colon, such as promotion of putrefaction due to low fiber intake. The PolyFermS intestinal platform has potential to be scaled down and adapted with multi-reactors to enhance screening efficiency.

To conclude, in the present study we showed the stability and reproducibility of PolyFermS continuous colonic fermentation models inoculated with immobilized elderly microbiota. Immobilization requires only small amounts of high quality fecal material to prime a gut model that can be stably operated over several months for testing parallel treatments in consecutive blocks [56]. The PolyFermS platform should be suitable for a range of *in vitro* gut microbiota investigations, from classical microbe interaction studies to complex ecological studies of the elderly gut microbiome investigated by in-depth analysis of the microbial diversity.

## Supporting Information

S1 Fig Correlations between genus-level phylogenetic groups and acetate and propionate of three last days at the end of the stabilization period of model 3. The correlations, assessed by Spearman are indicated by either red (positive) and blue (negative), the significant correlations (q < 0.05) are indicated by ‘+’. Only genus related phylotypes > 0.1% and with at least one significant correlation with metabolites are depicted.
(TIF)

S1 Table Primers used for enumeration of bacterial groups by qPCR. (DOCX)
