# Supplementary figures and images for: Design and Investigation of PolyFermS In Vitro Continuous Fermentation Models Inoculated with Immobilized Fecal Microbiota Mimicking the Elderly Colon
Source: PLoS One. 2015 Nov 11;10(11):e0142793. doi: 10.1371/journal.pone.0142793 (PMC4641611; doi:10.1371/journal.pone.0142793)

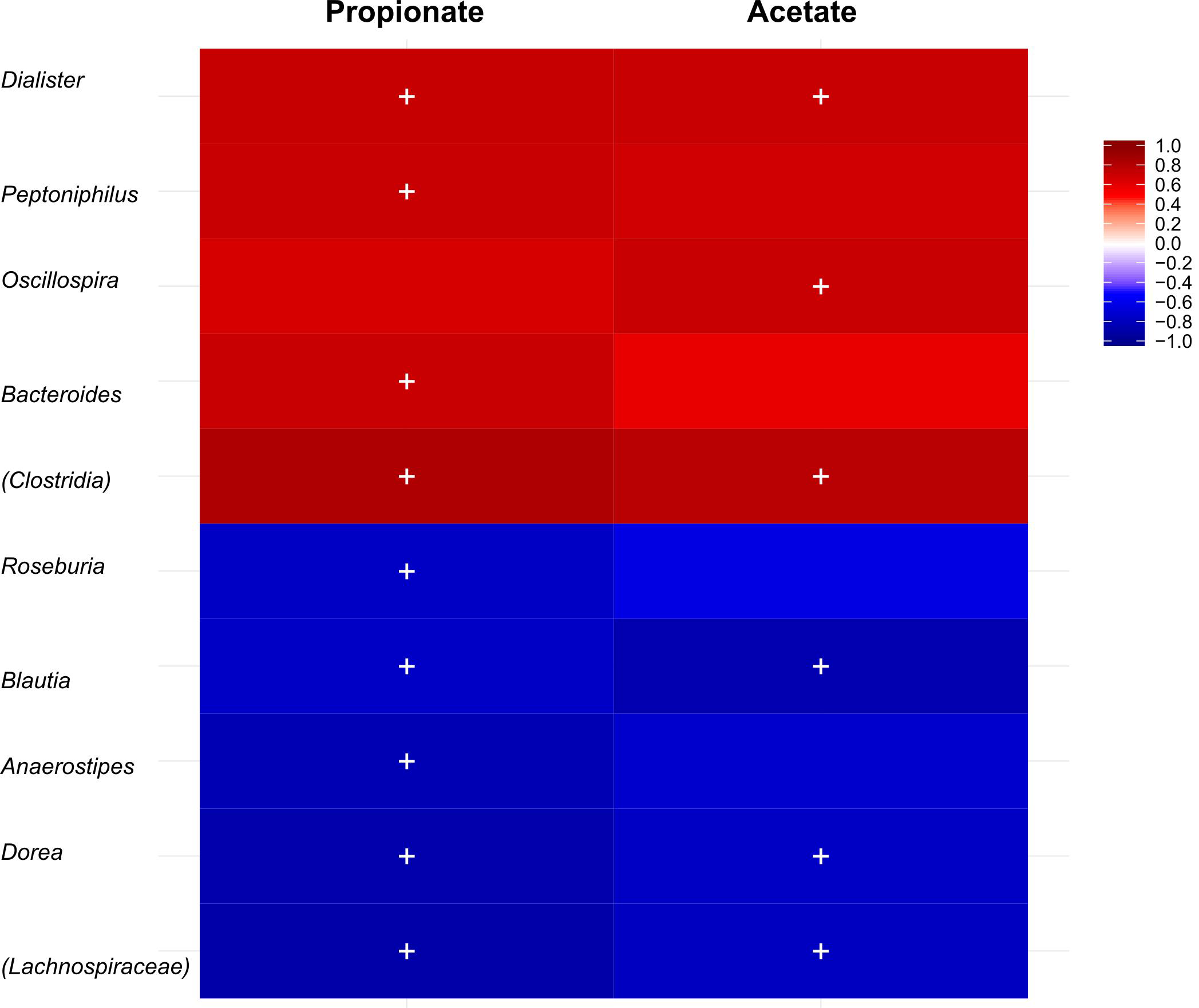

Supplement: S1 Fig — The correlations, assessed by Spearman are indicated by either red (positive) and blue (negative), the significant correlations (q < 0.05) are indicated by ‘+’. Only genus related phylotypes > 0.1% and with at least one significant correlation with metabolites are depicted. (TIF) [file pone.0142793.s001.tif]
