# Supplementary material for: Design and Investigation of PolyFermS In Vitro Continuous Fermentation Models Inoculated with Immobilized Fecal Microbiota Mimicking the Elderly Colon
Source: PLoS One. 2015 Nov 11;10(11):e0142793. doi: 10.1371/journal.pone.0142793 (PMC4641611; doi:10.1371/journal.pone.0142793)
Supplement: S1 Table — (DOCX) [file pone.0142793.s002.docx]

S1 Table. Primers used for enumeration of bacterial groups by qPCR.

| **Name** | **Sequence 5'-3'** | **Target gene/purpose** | **Reference** |
| --- | --- | --- | --- |
| Eub338F | ACTCCTACGGGAGGCAGCAG | Total 16S rRNA genes | (1) |
| Eub518R | ATTACCGCGGCTGCTGG |  |  |
| Bac303F | GAAGGTCCCCCACATTG | *Bacteroides* spp. | (2) |
| Bfr-Femrev | CGCKACTTGGCTGGTTCAG |  |  |
| RrecF | GCGGTRCGGCAAGTCTGA | *Roseburia* spp./*E. rectale* | (2) |
| Rrec630mR | CCTCCGACACTCTAGTMCGAC |  |  |
| Clep866mF | TTAACACAATAAGTWATCCACCTGG | *Clostridium* cluster IV | (2) |
| Clep1240mR | ACCTTCCTCCGTTTTGTCAAC |  |  |
| F_Lacto 05 | AGCAGTAGGGAATCTTCCA | *Lactobacillus/Pediococcus/Leuconostoc* spp. | (3) |
| R_Lacto 04 | CGCCACTGGTGTTCYTCCATATA |  |  |
| Fprau223F | GATGGCCTCGCGTCCGATTAG | *Faecalibacterium prausnitzii* | (4) |
| Fprau420R | CCGAAGACCTTCTTCCTCC |  |  |
| xfp-fw | ATCTTCGGACCBGAYGAGAC | *Bifidobacterium* phosphoketolase | (5) |
| xfp-rv | CGATVACGTGVACGAAGGAC |  |  |
| Eco1457F | CATTGACGTTACCCGCAGAAGAAGC | *Enterobacteriaceae* | (4) |
| Eco1652R | CTCTACGAGACTCAAGCTTGC |  |  |
| Met915F | AGGAATTGGCGGGGGAGCAC | *Methanobacteriales* | (6) |
| 1’100AR | TGGGTCTCGCTCGTTG |  |  |

**References to S1 Table**

1. Guo X, Xia X, Tang R, Zhou J, Zhao H, Wang K. Development of a real-time PCR method for Firmicutes and Bacteroidetes in faeces and its application to quantify intestinal population of obese and lean pigs. Lett Appl Microbiol. 2008;47(5):367-73.

2. Ramirez-Farias C, Slezak K, Fuller Z, Duncan A, Holtrop G, Louis P. Effect of inulin on the human gut microbiota: stimulation of *Bifidobacterium adolescentis* and *Faecalibacterium prausnitzii*. Brit J Nutr. 2009;101(4):541-50.

3. Furet JP, Firmesse O, Gourmelon M, Bridonneau C, Tap J, Mondot S, et al. Comparative assessment of human and farm animal faecal microbiota using real-time quantitative PCR. FEMS Microbiol Ecol. 2009;68(3):351-62.

4. Bartosch S, Fite A, Macfarlane GT, McMurdo ME. Characterization of bacterial communities in feces from healthy elderly volunteers and hospitalized elderly patients by using real-time PCR and effects of antibiotic treatment on the fecal microbiota. Appl Environ Microb. 2004;70(6):3575-81.

5. Cleusix V, Lacroix C, Dasen G, Leo M, Le Blay G. Comparative study of a new quantitative real-time PCR targeting the xylulose-5-phosphate/fructose-6-phosphate phosphoketolase bifidobacterial gene (xfp) in faecal samples with two fluorescence *in situ* hybridization methods. J Appl Microbiol. 2010;108(1):181-93.

6. Tymensen LD, McAllister TA. Community structure analysis of methanogens associated with rumen protozoa reveals bias in universal archaeal primers. Appl Environ Microb. 2012;78(11):4051-6.
